# On GPS spoofing of aerial platforms: a review of threats, challenges, methodologies, and future research directions

**DOI:** 10.7717/peerj-cs.507

**Published:** 2021-05-06

**Authors:** Shah Zahid Khan, Mujahid Mohsin, Waseem Iqbal

**Affiliations:** 1 College of Aeronautical Engineering (CAE), National University of Sciences and Technology (NUST), Islamabad, Pakistan; 2 Department of Information Security, National University of Science and Technology, Islamabad, Pakistan

**Keywords:** GPS spoofing, Security threats, Survey, Taxonomy, UAVs, Drones

## Abstract

Unmanned Aerial Systems (UAVs, Drones), initially known only for their military applications, are getting increasingly popular in the civil sector as well. Over the military canvas, drones have already proven themselves as a potent force multiplier through unmanned, round-the-clock, long-range and high-endurance missions for surveillance, reconnaissance, search and rescue, and even armed combat applications. With the emergence of the Internet of Things (IoT), commercial deployments of drones are also growing exponentially, ranging from cargo and taxi services to agriculture, disaster relief, risk assessment and monitoring of critical infrastructures. Irrespective of the deployment sector, drones are often entrusted to conduct safety, time and liability critical tasks, thus requiring secure, robust and trustworthy operations. In contrast, the rise in UAVs’ demand, coupled with market pressure to reduce size, weight, power and cost (SwaP-C) parameters, has caused vendors to often ignore security aspects, thus inducing serious safety and security threats. As UAVs rely on Global Positioning System (GPS) for positioning and navigation, they can fall prey to GPS jamming and spoofing attacks. The vulnerability of GPS to spoofing has serious implications for UAVs, as victim drones using civil GPS can be misdirected or even completely hijacked for malicious intents, as already demonstrated in several academic research efforts using commercially available GPS spoofing hardware. Beside UAVs, GPS spoofing attacks are equally applicable to other GPS-dependent platforms, including manned aircraft, ground vehicles, and cellular systems. This paper conducts a comprehensive review of GPS spoofing threats, with a special focus on their applicability over UAVs and other GPS-dependent mobile platforms. It presents a novel taxonomy of GPS spoofing attacks and critically analyzes different spoofing techniques based upon placement of spoofing device, attack stealthiness, attack methodologies, and objectives of the attacker. We also discuss some of the recent experiments from open literature which utilized commercially available hardware for successfully conducting spoofing attacks.

## Introduction

Drones are becoming increasingly popular having multifaceted roles for both commercial and military applications. Some estimates suggest that, at present, more than 10,000 drones are serving world-wide as high bandwidth mobile data backbones, security surveillance, rescue services, autonomous air taxis, and relief operations (Guvenc et al., 2018; Wesson & Humphreys, 2013). Moreover, the drone market value has been estimated to reach 1.85 billion USD by the year 2024 (Nassi et al., 2019). In the military sector, they are used for surveillance, tracking and delivery of armed payload. Nowadays unmanned air vehicles (UAVs) are also being employed in combat and can carry various missiles, like the “MQ-8B Fire Scout” used by USA (Wesson & Humphreys, 2013). Even new fighter jets have been converted and used as fully autonomous UAVs (Nacouzi et al., 2018).

Modern-day UAVs rely heavily on Global Navigation Satellite System (GNSS) for Guidance, Navigation and Control (GNC). Among the available GNSS options, Global Positioning System (GPS) is the most common and widely used satellite navigation system. The autonomous UAVs are even more dependent on the flight aids such as the autopilot and navigational and dynamic-positioning. In addition to its celebrated accurate positioning service, GPS also offers time synchronization with the precision of about 10 billionth of a second using the on-board atomic clocks (Wei & Sikdar, 2019). Time-sensitive systems such as synchrophasors in power grid systems use GPS time for a synchronous state estimation and offline engineering analyses (Shepard et al., 2012). All these systems are designed assuming the trustworthiness of the GPS services (Bhatti & Humphreys, 2017).

GPS-dependent UAVs require accurate, trustworthy and uninterrupted position information for their safe operations. However, different research efforts have shown that GPS signals can be jammed or spoofed owing to its inherent vulnerabilities. Because of the low signal power (around −130 dBm), the GPS services can easily be disrupted through the transmission of high power jamming signals directed towards the victim platform (Arteaga et al., 2019). Besides, the civil GPS services have no encryption or authentication mechanisms and therefore, the satellite signals can easily be replicated or fabricated, which can subsequently be utilized for launching sophisticated GPS spoofing attacks.

GPS spoofing is the act of replicating or falsified production of the GPS signals to deceive a targeted GPS unit or receiver in particular, manipulating its Position, Velocity and Timing (PVT) parameters (Psiaki & Humphreys, 2016). With the emergence of low-cost user tunable Software Defined Radios (SDRs) and online open source projects and tutorials for hobbyist and newbies, launching of GPS spoofing attacks against UAVs have become practical, calling for stronger built-in spoof-resilient measures, in particular for safety of mission-critical airborne applications (Jafarnia-Jahromi et al., 2012; Huang & Yang, 2015; Wang, Chen & Pan, 2015).

A successful GPS spoofing attack may have dangerous consequences as it can divert the course of the flight or can cause a drone to crash (Dulo, 2015). Various research efforts (Seo et al., 2015; Noh et al., 2019) conclude that a GPS guided drone can be forced to deviate from its course, or even hijacked, if its current position and intended travel path is known to the attacker. Through spoofing, the safety feature of “Geo-fencing” can also be bypassed and thus the targeted drone can be made to violate no-flying zones (Schmidt, 2015). This vulnerability can be exploited by drug smugglers and others to trespass controlled borders across prisons for drug trafficking and illegal surveillance (US National PNT Advisory Board, 2018). A military-grade armed UAV could cause a catastrophe if the machine somehow gets hijacked and ultimately used by a terrorist organization.

The vulnerability of civil GPS to spoofing attacks was first demonstrated in an unclassified test exercise “GYPSY” by Department of Homeland Security (DHS) on 19 June 2012 at White Sands Missile Range (WSMR) (Shepard et al., 2012). During that exercise, a GPS spoofing attack against “Hornet”, a mini-drone, was carried out at a height of 40*feet*, resulting in manipulating its perceived position and time. Another major GPS spoofing claim against military grade UAV was made by Iranian Army (Hartmann & Steup, 2013), when a US RQ-170 Sentinel drone was successfully captured. However, the authenticity of the claim and exact circumstances of the UAV capture are unverified and controversial. In 2016, another incident of UAV deception through GPS spoofing attack, was reported in which a US custom bureau’s UAV was targeted by Mexican drug dealers and traffickers (Khan, Brohi & Jhanjhi, 2020). Moreover, similar GPS based spoofing attacks have also been demonstrated in several other works (Dey et al., 2018; Horton & Ranganathan, 2018; Arteaga et al., 2019; He & Qiao et al., 2019; He & Liu et al., 2019; Ma et al., 2020; Zheng & Sun, 2020) against Hornet Mini, DJI’s Matrice 100, Phantom 3 and 4 Pro, 3DR Solo, Parrot’s AR Drone 2.0 and Bebop 2 drones.

The applicability of GPS spoofing attacks against GPS-dependent non-aerial platforms such as delivery trucks, maritime craft, smartphones, road navigation systems, and commercial GPS receivers have also been extensively evaluated (Warner & Johnston, 2002; Huang & Yang, 2015; Wang, Chen & Pan, 2015; Bhatti & Humphreys, 2017; Zeng et al., 2017; Horton & Ranganathan, 2018; Zeng et al., 2018; Cao, Luo & Liu, 2019; Goavec-Merou, Friedt & Meyer, 2019; Gaspar et al., 2020; Rustamov et al., 2020). Similarly, attacks against GPS-time dependent systems such as smartwatches, smart-grid time reference receivers, CDMA phone towers and Network Time Protocol (NTP) servers have also been studied in literature (Shepard et al., 2012; Wang, Chen & Pan, 2015; Humphreys, 2015; Karit, 2017).

### Survey’s rationale and methodology

Considering the growing research interests and practical contributions towards GPS spoofing of cyber-physical systems, a requirement exists to comprehensively analyze the emerging threat landscape and logically group these threats based on diverse spoofing techniques, multifaceted attack variables, evolving attack objectives, and corresponding countermeasures. Existing surveys on GPS spoofing (Jafarnia-Jahromi et al., 2012; Günther, 2014; Sathyamoorthy, 2015; Krishna & Murphy, 2017) are outdated and do not fully cover the state-of-the-art. Moreover, these efforts focus only on a confined subset of the holistic threat landscape posed by GPS spoofing. In particular, existing surveys on threats to aerial platforms, such as presented by Nassi et al. (2019), are generic in nature and do not cover the details of GPS spoofing threats. Moreover, the available literature also lack a proposal to comprehensively classify existing techniques in the form of a taxonomy with an aim to facilitate and steer further research in focused domains. This effort seeks to conduct a detailed review of UAVs’ dependency over GPS and present a cohesive and novel taxonomy of GPS threat variables, goals, and trade-offs, while focusing on aerial platforms.

**Figure 1:**
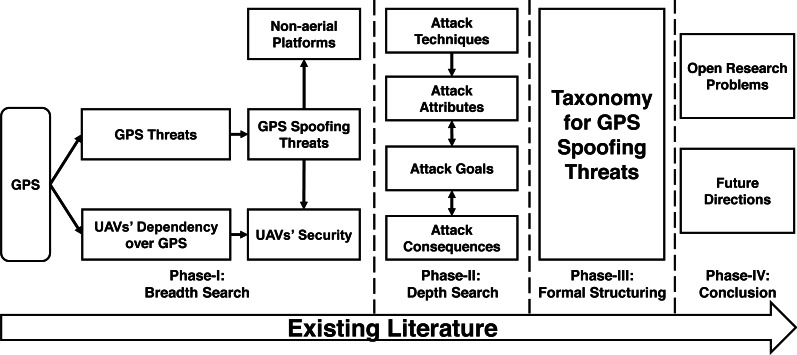
Survey’s methodology.

The overall survey approach followed by this work is summarized in Fig. 1. Our survey follows a semi-systematic methodology (Snyder, 2019), which explores and narrows-down the relevant literature in multiple phases. The first phase of the literature review focuses on a breadth search within the broad domains of GPS security threats, in general and spoofing threats, in particular. In parallel, this phase categorizes drones with reference to their dependencies over GPS as this factor further assists in evaluating threat levels, applicable techniques, and impact of GPS spoofing attacks over drones. While the prime focus of our survey revolves around drone-specific spoofing attacks, we have also covered the breadth of similar efforts against non-aerial platforms to serve as a ready reference and perform comparative analysis of variation in attack parameters in both of these scenarios. Therefore, segregation of existing GPS spoofing attempts against non-aerial platforms is also performed during Phase-I. Phase-II conducts a depth search to shortlist and group existing literature within the domain of GPS spoofing. During this phase, we conduct a comprehensive analysis of existing research contributions based on the techniques deployed for GPS spoofing, including attack attributes, goals, and consequences. The analyses performed in Phase-II is formalized as a taxonomy in Phase-III, which structures the inherent relationships among complex spoofing parameters and logically aligns them for better understanding. The inferences drawn after evaluating the existing literature in line with the proposed taxonomy are summarized in Phase-IV with pointers to open research problems and future directions.

### Targeted audience

The analyses presented by this paper can benefit a wider research and development community, working in the domain of GPS-driven GNC applications, both for offensive and defensive purposes. A better understanding of existing GPS spoofing threats, as classified by our work, can help in neutralizing rogue UAVs as well as defending friendly UAVs against spoofing attacks.

### Paper organization

The rest of the article is organized as follows. In ‘Background’, we present an overview of the GNSS in general and GPS, in particular. ‘Drones GNC Dependency and Allied Threats’ provides a discussion on the dependency of drones over GPS. We analyze the GPS based threats to the dependent systems, specifically UAVs, in ‘GPS Threat Landscape’. ‘Taxonomy of GPS Spoofing Attacks’ presents a novel taxonomy of GPS spoofing attacks based on spoofer placement, stealthiness, attack technique, and objective of the attacker. Challenges and limitations of GPS spoofing of static targets as compared to moving and aerial platforms are featured in ‘Spoofing Challanges’. ‘Open Problems and Future Research Directions’ discusses some of the open problems identified by this work to steer future research directions. Finally, ‘Conclusion’ concludes the paper.

## Background

For readers not familiar with relevant terminologies, this section provides the fundamental background knowledge of Global Navigation Satellite System (GNSS) and location measurement process using GPS.

### Global Navigation Satellite System (GNSS)

GNSS is an umbrella term used for satellite Positioning, Navigation and Time (PNT), provided by satellite signals transmitted from space. To avoid dependency, several countries operate their independent GNSS systems with varying degrees of coverage and operational capabilities, including GPS by the USA, GLONASS by Russia, Galileo by the European Space Agency, and BeiDou Navigation Satellite System (BDS) by China. The four major systems have distinct carrier frequencies and they also employ different modulation schemes (Maksutov et al., 2019). As an example, some of the GLONASS signals use Frequency Division Multiple Access (FDMA) modulation scheme, while the GPS uses Code Division Multiple Access (CDMA). However, the newer versions of the GLONASS are also using CDMA as a modulation scheme. Despite having individual characteristics, all these systems deploy a similar principle of operation and are designed to serve a common goal, which is broadcasting a radio frequency signal with a precise time-stamp, enabling the users to receive and decode these signals to determine their position (Ioannides, Pany & Gibbons, 2016). A GNSS receiver calculates its position and time by the principle of “trilateration. To have a 3-dimensional location-fix and time synchronization, navigational data from at least four satellites in the constellation is needed (Larcom & Liu, 2013). All the currently operational GNSS systems including GPS offer no encryption or source authentication for services available for public use (Ioannides, Pany & Gibbons, 2016), making them equally susceptible to attacks discussed later in ‘GPS Threat Landscape’. Recently, some GNSS service providers have introduced spoof resistant services, like Galileo’s Open Source Navigation Message Authentication (OSNMA), which enables authentication of navigational data on Galileo. NMA validates the received GNSS signal, making it robust against GNSS spoofing attacks. However, wide scale implementation of this authentication service would require updating the firmware of existing Galileo receivers. Another limitation is that NMA does not currently offer authentication service for the ranging measurements (Gutierrez, 2020).

### Global positioning system (GPS)

Originally known as the NAVSTAR, GPS was launched in 1978 only for the US military. However, later in 1994, GPS services were made fully available to the rest of the world. GPS has emerged as the de-facto GNSS standard due to its global coverage, wide adoption and acceptability.

**Figure 2:**
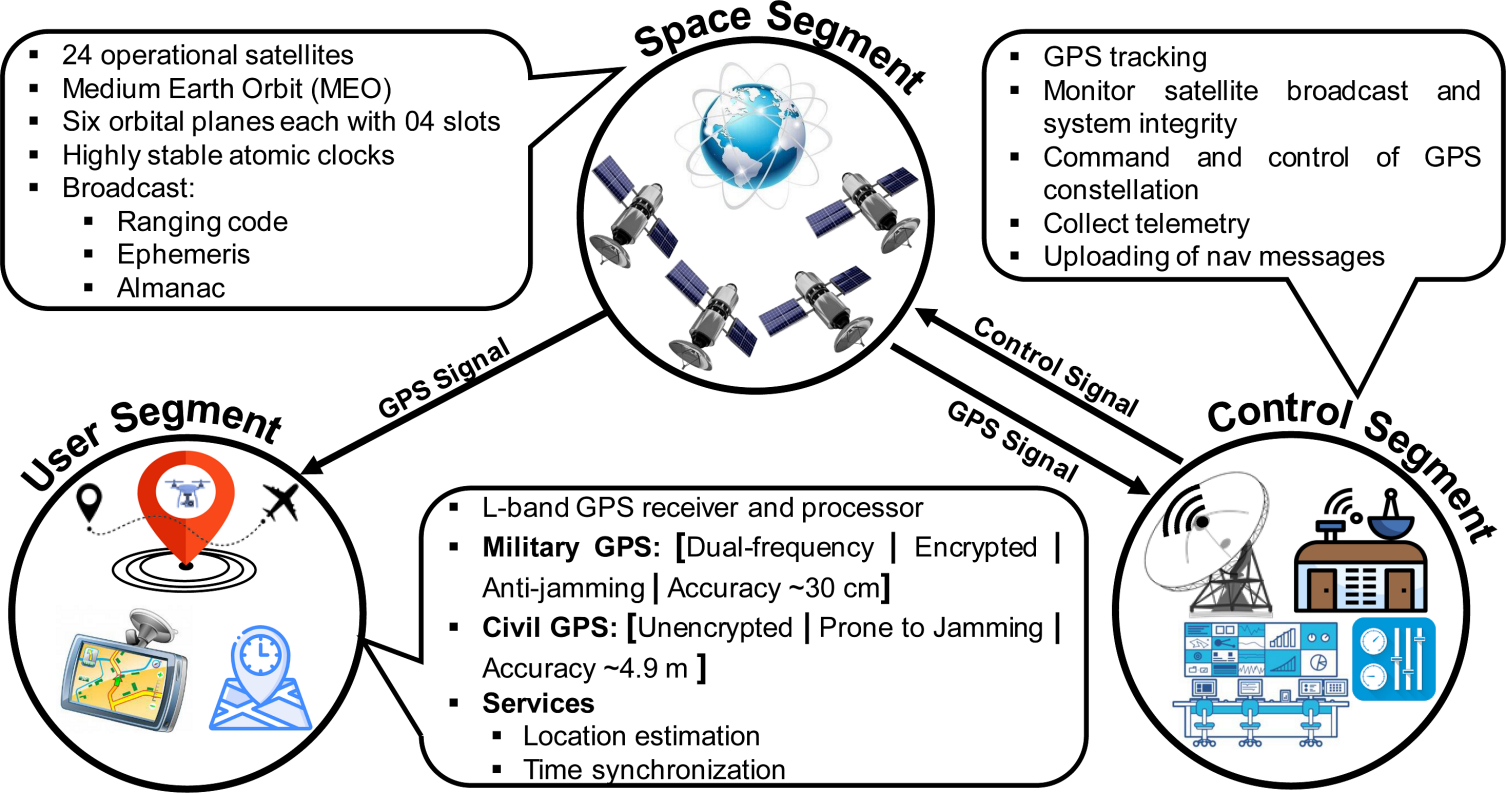
GPS Segments.

#### GPS segments

The system’s architecture of GPS, as of any other GNSS, can be segregated into three main domains, known as Space segment, Control segment, and User segment as shown in Fig. 2.

• **Space segment:** This segment contains a constellation of satellites that broadcast Radio Frequency (RF) signals containing coded information and navigational data for PVT estimation at the user end. GPS has 24 operational satellites in Medium Earth Orbit (MEO), 22,200 km above the surface of the Earth (NCO Space-Based PNT, USA, 2021b). These satellites revolve around the earth in six equally-spaced orbital planes (four slots in each orbit), managed at an inclination of 55 degrees with reference to the earth’s equator.
• **Control segment:** The control segment is tasked with monitoring and ensuring the integrity of the GPS by exercising command and control over the GPS constellation. It consists of a global network of ground facilities that collects telemetry to monitor and analyze the broadcast signal and sends commands and uploads navigation messages when required.
• **User segment:** The user segment refers to a diverse range of user GPS receivers and associated services, both military and civil, which can receive and decode the information broadcasted by satellites for position and time estimation. The GPS receiver is equipped with an L-band receiver and processor which performs positioning and time computations for supporting overlaying user applications.

#### GPS transmission

**Figure 3:**
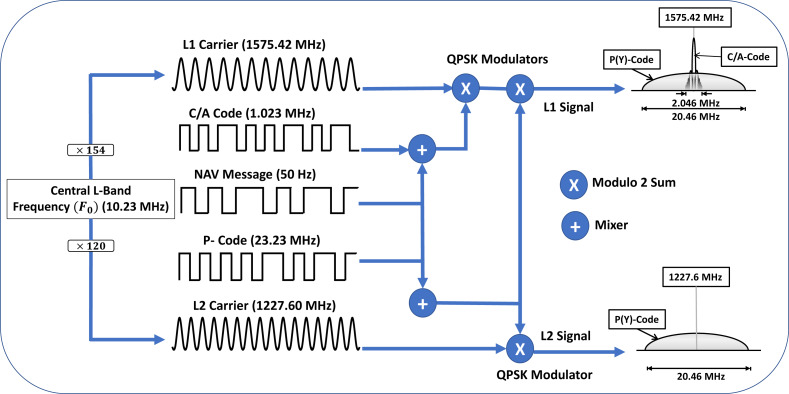
GPS signal generation and composition.

The signal generation and modulation process followed by the GPS Space segment is illustrated in Fig. 3. The GPS satellites generate a central L-band frequency (*F*_0_) of 10.23 MHz, using its on-board atomic clock. This base frequency is multiplied by 154 and 120 to generate the two carrier frequencies, L1 at 1575.42 MHz and L2 at 1227.60 MHz, which are subsequently modulated by Coarse Acquisition Code (C/A) and Precise (P) ranging codes: a combination of the data message and a unique code, to produce a spread spectrum signal of 2.046 MHz and 20.046 MHz bandwidth respectively (Tamazin, Karaim & Noureldin, 2018). Each satellite has a unique Pseudo-Random Noise (PRN) code that is nearly orthogonal to each other, which helps the receiver to differentiate between each satellite in the GPS constellation, improve Signal to Noise Ratio (SNR), ensure accurate ranging, and enhance robustness against signal interference (Jan Van Sickle, 2021).

The P(Y) is an encrypted signal for the military, with a claimed accuracy of few centimetres. The C/A signal has an accuracy of 4.9 meters and is freely available for public use without any embedded encryption or authentication (NCO Space-Based PNT, USA, 2021a). However, its accuracy and availability is affected by various factors, such as the urban canyon, trees, building and other obstacles. GPS transmits the following types of data (Schmidt et al., 2016):

• A ranging code, enabling the receiver for computing PVT solution.
• Ephemeris data containing location information of individual satellites.
• Almanac data having the orbits, locations and status information for all the satellites in the constellation.

The navigation message, which is a key component of the transmitted signal contains information regarding the ephemeris, almanac and clock bias parameter (Karaim et al., 2013).

#### GPS reception

At the User segment end, the GPS signal is received by an antenna attached to the RF front-end of the GPS receiver. At the front-end, the weak GPS signal is filtered, amplified, digitized and converted to the baseband signal (Tamazin, 2015). The output of the front-end is then processed by a signal processing unit for calculating navigational information. At this stage, the receiver extracts the pseudo-range and its rate of change information independently for all the satellites in view, for subsequent estimation of the PVT solution. This process involves the stages of Acquisition, Tracking, Monitoring, Extraction, Measurement Generation and PVT solution for navigational requirements (Tamazin, Karaim & Noureldin, 2018).

#### GPS working: an overview

A GPS receiver uses satellite’s location information and signal’s delay for calculating its position and time synchronization. The signal received by the GPS receiver contains information regarding the satellite’s position and time as a GPS time-stamp. A local copy of the received signal code is generated by the receiver for comparing it with the received signal to calculate the clock error and pseudo-range (*R*). To have a 3-dimensional location-fix and time synchronization, navigational data from at least four satellites in the constellation is needed to satisfy the trilateration equation. Mathematically:

(1) \begin{eqnarray*}R=d+\Delta \exists \Delta =c.\delta \end{eqnarray*}

Where *d* is the range, *c* represents the speed of light and Δ is the offset in range due to local clock error (*δ*) of the GPS receiver. The 3D location of the GPS receiver can be computed using the following set of four equations.

(2) \begin{eqnarray*}{\forall }_{1\leq i\leq 4} (X-{x}_{i})^{2}+(Y-{y}_{i})^{2}+(Z-{z}_{i})^{2}=({R}_{i}-\Delta )^{2}\end{eqnarray*}

Where (*X*, *Y*, *Z*) and (*x*_*i*_, *y*_*i*_, *z*_*i*_) indicate the position of GPS receiver and the *i*th satellite, respectively. Equation (2) has four unknowns: 3-dimensional location coordinates of the GPS receiver and time offset. A 3D-position (*X*, *Y*, *Z*) and local clock error (*δ* = Δ∕*c*) can be derived by the receiver after solving these four equations, one for each satellite (Larcom & Liu, 2013).

## Drones Gnc Dependency and Allied Threats

An Unmanned Aerial Vehicle (UAV) or a drone is a small aerial platform that can be controlled remotely and is recognized as an aircraft by the International Civil Aviation Organization (ICAO) (Arteaga et al., 2019). All UAVs require a sensory system for guidance and control systems that enables them to navigate their mission. In simple terms, instructions are generated by the guidance system for UAVs’ trajectory and mission execution (Elkaim, Lie & Gebre-Egziabher, 2014). Traditionally, the radio control, and autopilot executes the Guidance, Navigation, and Control (GNC) of the drone (Hassanalian, Radmanesh, and Ziaei-Rad, 2012). Generally, control of drones over short distance results in a negligible lag and high bandwidth with minimal losses, while control over thousands of miles results in significant lag in control, low bandwidth, and considerable losses (Hassanalian & Abdelkefi, 2017). Therefore, UAVs capable of long distance and endurance flight are typically augmented with autopilot features, capable of stabilizing flight and performing various autonomous functions in case of loss of the Command and Control (C2) link. The autonomy level of the drone is proportional to its GNC capabilities.

### Drone’s operational modes

Modern-day UAVs leverage a wide range of sensors, including GNSS, for their positioning, orientation, path profiling, guidance, and navigation. Apart from GNSS, some of the other sensors which are typically leveraged by drones/missiles include Inertial Measurement Unit (IMU), TERrain COntour Matching (TERCOM), accelerometer, magnetometer, gyroscope, and barometer. However, these non-GNSS based GNC systems are considered beyond the scope of our work. Among different GNSS, GPS is the most widely deployed system due to its wide acceptability and free global coverage. Drone’s dependency over GPS is subjected to the level of autonomy, targeted application, and flight mode. The various “Flight Modes” of modern drones, such as ArduPilot Dev Team (2020), can be grouped under three broader operational categories: Manual, Semi-Autonomous Assisted, and Autonomous. Table 1 maps key flight modes of drones with corresponding operational mode, each having a varying dependency over GPS-guidance and therefore, presents different level of threat exposure. A brief introduction of these operational modes is given below (Mulas, 2016; Hassanalian & Abdelkefi, 2017).

**Table 1:** Drone’s operational modes.

Operational Mode	Range	Example Flight Modes	GNC Dependency	GPS Threats
Manual	VLOS	Manual	C2 Link	No
Semi-Autonomous Assisted	EVLOS	Stabilize, Alt Hold Circle, Drift, Follow, Loiter, Zig Zag, RTL	C2 Link GPS	Yes
Automatic	BVLOS	Auto, Guided, Smart RTL	GPS	Yes

#### Manual mode

In *manual mode*, drones are regulated all the time through a Remote Control (RC) usually known as telemetry, within Visual Line Of Sight (VLOS) and do not require GPS for guidance, though this mode requires technical skills on part of the operator to control the aircraft. Since GPS is never used in the *manual mode*, drones in this mode are not vulnerable to GPS-based threats. However, manually operated drones can still be subjected to those threats which target air to ground or air to air (e.g., slave drone in a swarm) C2 links.

#### Semi-autonomous assisted

Drones in *semi-autonomous assisted mode* are also governed by a ground operator, with assistance from the autopilot, constituting various sensors including GPS. As an example, various automated flight modes of ArduPilot (https://ardupilot.org/plane/docs/flight-modes.html), a widely used open source auto pilot system, such as *circle*, *drift*, *follow*, *loiter*, *zig zag* and *return to launch (RTL)*, use GPS for executing commands and fall under semi-autonomous category. Other similar functions like *stabilize*, *alt hold* and *land* make use of additional connected Micro Electro-Mechanical Systems (MEMS) sensors like altimeter, accelerometer and other vision-based sensors. These commands can be manually relayed to the drone while operating in *semi-autonomous mode*. In such a case, drones are dependent on both the C2 link and the GPS for GNC services and can operate in Extended Visual Line Of Sight (EVLOS).

#### Autonomous

In *autonomous mode*, the on-board Autopilot is provided with a flight plan e.g., *guided*, *auto* and *smart RTL* modes of the Ardupilot. After this mode is activated the ground controller cannot (or is not required to) intervene for the control. The aircraft requires no user input and is solely dependent on the integrated guidance system including obstacle avoidance and course rerouting, in case of *smart RTL mode*. In a GPS guided drone, PVT solution is calculated for navigating course and execution of mission Beyond Visual Line Of Sight (BVLOS). Since the C2 link is never/rarely used in the autonomous operational mode, the threat vectors are restricted to GPS-based threats only.

### Mode-specific UAV threat landscape

UAVs in *manual mode*, being unsusceptible to GPS-based threats, are vulnerable to RF link attacks (Pleban, Band & Creutzburg, 2014; Costello, 2017; Dey et al., 2018; Arteaga et al., 2019; Nunez, Tran & Katangur, 2019; Yihunie, Singh & Bhatia, 2020). Such cyber-attacks primarily utilize drone’s C2 links and implementation-specific software, hardware, and networking vulnerabilities. Manesh & Kaabouch (2019) surveyed the cyber-attacks on drones and proposed three broad categories of these attacks as: (1) Data Interception, (2) Data Manipulation, and (3) Denial of Service. These attacks attribute to C2 link, Automatic Dependent Surveillance–Broadcast (ADS-B) and Navigation data received by the drone. Some of the network/C2 link based attacks are briefly discussed below:-

• **Compromised Network Ports:** The vulnerable network ports like FTP (Port 21) and Telnet (Port 23) can be used for gaining access to the software root directories of various consumer drones such as DJI Phantom 4, Parrot Bebop 2 and AR Drone 2.0, allowing the attacker to cause physical damage to the drone (Pleban, Band & Creutzburg, 2014; Arteaga et al., 2019). Here, the network ports may be regarded as software level vulnerable entry points to inject attack vectors and compromise the system.
• **Denial of Service (DoS):** Arteaga et al. (2019) discussed that flooding of WiFi based C2 links with certain specific packages may result in DoS, thus causing disruption of manual control over drones.
• **De-authenticating Controller:** A de-authentication attack aims to disconnect the established link between the controller and the drone by snooping into the communication link and then sending multiple de-authentication packets. Several research efforts (Dey et al., 2018; Nunez, Tran & Katangur, 2019; Yihunie, Singh & Bhatia, 2020) presented the effectiveness of de-authentication attacks against Parrot’s Bebop 2, Mambo FPV and AR Parrot 2.0 drones using aircrack-ng (https://www.aircrack-ng.org/): an open source penetration testing tool, resulting in hijacking of the drone. Furthermore, Maldrone, an airborne malicious drone that uses another such open source software package SkyJack (https://samy.pl/skyjack/), utilizes similar technique for independently launching network de-authentication attacks for taking control over the target.
• **Spoofing IP and MAC Addresses:** This network cyber-attack is also applicable to C2 links of drones. Costello (2017) demonstrated this attack against Parrot AR Drone 2.0 by creating the network IP alias and spoofing MAC address of the primary controller, gaining complete control of the target.

In *semi-autonomous assisted mode*, drones have added dependency over the GPS and are prone to both C2 link and GPS-based attacks. Compromise of any of these technologies by an attacker may lead to complete control of the targeted drone. GPS-dependent flight modes, such as *follow*, *loiter*, and *RTL* are mostly independent of the user input through C2 link and take reference primarily from the GPS. Finally, the *autonomous mode* operations of drone are susceptible to GPS-driven attacks only since the mission profiling and execution in this mode rely only upon GPS-based guidance and control.

## GPS Threat Landscape

In addition to the logical attack vectors through the C2 link as discussed in the previous section, the on-board GPS can also serve as an unguarded attack entry point due to the inherent vulnerabilities of civil GPS. Interrupting the GPS signal can lead to DoS, errors in PVT measurements or reporting of falsified location and time information by the receiver. Irrespective of the intention of the emitter, any GPS interference signal can be considered as a variant of either jamming or spoofing, as also categorized by Fernandez-Hernandez et al. (2019). Jamming leads to DoS that may affect continuum and availability of guidance signals; whereas, spoofing may result in violations in data integrity if the malicious signal is considered as valid by the receiver.

This section introduces the two main categories of GPS-based threats (i.e., Jamming and Spoofing) and broadly defines the sub-categories of GPS spoofing attacks, to build a foundation for our survey. Followed by this broader classification, the next section presents a comprehensive taxonomy of GPS spoofing, while integrating the discussions on relevant research efforts from existing literature falling within the categories defined by the taxonomy. The overall summary and critical analysis of relevant literature covered by our survey is given in Table 2. Our work extensively reviews open literature on GPS spoofing and initially groups these works based on targeted (victim) systems as (a) Drones, (b) Non-aerial Platforms, and (c) Time Spoofing attacks. After this broader classification, each of the covered efforts is analyzed based upon the type of spoofing equipment used, spoofer’s portability, attack’s sophistication, stealthiness, and range. Additionally, the key limitations of these attacks, as perceived by our analysis and supporting literature, is also summarized in the mentioned table.

**Table 2:** Summary and analysis of existing efforts towards GPS spoofing.

Reference	Target	Spoofing system	Device placement	Sophistication	Stealthiness	Limitations
**Location spoofing - drones**						
Shepard et al. (2012)	Hornet [Mini Drone]	SDR [With custom made DSP core] (Humphreys et al., 2008)	Off-board	Sophisticated	Covert	(1) Victim’s position required. (2) Fixed distance between attack device and victim.
Dey et al. (2018)	DJI [Phantom 4 Pro]	GPS Simulator [LabSat3]	Off-board	Simplistic	Covert	GPS denied environment.
He et al. (2019)	Parrot [AR Drone 2.0]	SDR [HackRF One]	Off-board	Intermediate	Overt	(1) Attack requires precise location information of the target. (2) Target was in Loiter mode/ hovering at 10 *m*.
Horton & Ranganathan (2018)	DJI [Matrice 100]	SDR [BladeRFx40]	Off-board	Simplistic	Overt	GPS denied environment.
Arteaga et al. (2019)	3DR [Solo]	SDR [BladeRFx40]	Off-board	Simplistic	Overt	Limited attack range.
He et al. (2019)	Drone	SDR [HackRF One]	Off-board	Simplistic	Overt	Attack could have been detected by analyzing drone camera feed.
Ma et al. (2020)	DJI [Phantom 3 SE]	Custom Designed	Off-board	Intermediate	No Information	(1) Required real-time location information of the target. (2) *Autonomous Mode* only, attack.
**Location spoofing - non-aerial platforms**						
Warner & Johnston (2002)	Truck [Navigation System]	GPS Simulator [WelNavigate GS720]	Off-board	Simplistic	Overt	Limited attack range.
Bhatti & Humphreys (2017)	Yacht	Custom Designed Kerns et al. (2014)	On-board	Intermediate	Partially Covert	Required physical access to the target.
Huang & Yang (2015)	Smartphone [Nexus 5]	SDR [BladeRFx40]	Off-board	Simplistic	Overt	Limited attack range.
Wang, Chen & Pan (2015)	Smartphone [iPhone 6]	SDR [BladeRFx40]	Off-board	Simplistic	Overt	Limited attack range.
Silva (2017)	Smartphone [Android]	SDR [HackRF One]	Off-board	Simplistic	Overt	Limited attack range.
Zeng et al. (2018)	Car [Navigation System]	SDR [HackRF One]	On-board [Limpet]	Simplistic	Overt	(1) Perceived victim’s route information. (2) Required physical access to the target.
Horton & Ranganathan (2018)	Smartphone [HTC Desire 626]	SDR [BladeRFx40]	Off-board	Simplistic	Overt	Device was disconnected from the Internet.
Goavec-Merou, Friedt & Meyer (2019)	(1) Smartphone [Android] (2) Ublox-NeoM8T	SDR [Pluto]	Off-board	Simplistic	Overt	System’s configuration was valid for only a few hours.
Gaspar et al. (2020)	(1)Android Phone (2) U-blox GPS receivers	SDR-Custom Designed [BladeRF Based]	Off-board	Simplistic	Overt	Limited attack range.
Rustamov et al. (2020)	(1) Smartphone [Android] (2) U-blox receiver	SDR [HackRF One] based GPS spoofer	Off-board	Simplistic	Overt	Limited attack range.
**GPS based time spoofing**						
Shepard et al. (2012)	Smart-grid [Time-reference GPS receiver]	SDR-Custom Designed [With DSP core]	Off-board	Sophisticated	Covert	Perceived victim’s position information.
Wang, Chen & Pan (2015)	Smartwatch [Apple]	SDR [BladeRFx40]	Off-board	Simplistic	Overt	Limited attack range.
Karit (2017)	NTP server	SDR [BladeRF]	Off-board	Simplistic	Overt	Moving time >5 min results in NTP demon shut down.

### Jamming

Jamming is the generation and transmission of enough power signal in the direction of a target that may cause the GPS receiver of the victim unable to track the original GPS signal (Gerdan, Coombe & Takac, 1995). It is a very common and highly undesirable real threat. Westbrook (2019) discussed about new geographies of conflict due to the prevailing use of military-grade GPS jamming equipment by both state and non-state actors as an offensive tool of electronic warfare. Owing to the GPS signal being very weak and well below the background RF noise level measured by the receiver (Silva, 2017; Van den Bergh & Pollin, 2019), it can be affected by the ionospheric attenuation and other factors such as the path followed by the satellite’s transmissions to the receiver and unlicensed use of the GPS band. The jamming of a GPS signal depends upon various parameters such as the power of the original signal and distance of the jamming signal generator from the GPS receiver etc. There are two main characteristics of the jamming signal, the “central frequency” and “Jamming to Signal ratio (*J*∕*S*)” measured in dB. Jamming signal overpowers the signal strength of the authentic signal, described by “Carrier to Noise Density Ratio (*C*∕*N*_*o*_)”, which is a fundamental signal quality parameter for a GPS receiver (Hofmann-Wellenhof, Lichtenegger & Wasle, 2008). When the jamming equipment is brought closer to the GPS receiver, the power of the jamming signal increases, thus reducing the effective signal strength of the original signal (Silva, 2017).

Regarding UAVs, with a firmware-coded safety mode, an attacker can force a drone to land when both the GPS signal and C2 link are jammed (Tedeschi, Oligeri & Di Pietro, 2020). In 2012, a small UAV resulted in a crash due to GPS jamming, costing a human life (Krishna & Murphy, 2017). In the literature, several works explored the effects of jamming on UAVs. Sathyamoorthy et al. (2020) evaluated the effect of GPS jamming signal on two different UAVs using different jamming signal power levels. The authors concluded that the military GPS signal shows robustness against the jamming signal, while the civil GPS signal is more susceptible to jamming. Further detailed discussion of jamming and related RF interference affecting the GPS signal have been explored by Fernandez-Hernandez et al. (2019) and Gerdan, Coombe & Takac (1995). In particular, Fernandez-Hernandez et al. (2019) categorized the GPS jamming threats into four types based on objectivity of exposure and sophistication of attack; whereas, Gerdan, Coombe & Takac (1995) presented three different case studies of the adverse effects of jamming on GPS signal’s acquisition. Furthermore, Medina et al. (2019) explored the effects of GNSS jamming, including GPS, from a maritime navigation’s perspective.

GPS jamming can be performed using a diverse range of hardware (Karit, 2017), while employing a variety of jamming methods and techniques. Elezi et al. (2019) investigated multiple jamming methods through software simulations and concluded that Spot Noise Jammers, being highly effective against the GPS L1 band, caused the highest Bit Error Rate (BER).

For mitigating the effects of jamming on UAV, Tedeschi, Oligeri & Di Pietro (2020) presented an anti-jamming navigational algorithm by leveraging the jamming signal for localization of the source through Received Signal Strength (RSS). The proposed approach requires considerable software and hardware upgradations for implementing anti-jamming features, including an on-board SDR for jammer’s localization and a custom-built firmware. Moreover, the offered solution is not effective against mobile or adaptive jammer. Seferoglu & Turk (2019) investigated specific waveforms of some commercially available GPS jammers and presented discussion into anti-jamming solutions.

### Spoofing

Compared to GPS jamming, the spoofing threat is often pronounced as more dangerous as a spoofer can lead the target to produce erroneous PVT solution or even gain complete control over GPS-driven drone’s trajectory by re-radiating or fabricating counterfeited GPS signals (Horton & Ranganathan, 2018).

GPS spoofing is a more challenging and technology-intensive operation as compared to brute-force jamming since a failed spoofing attempt can still yield the desired or unintentional jamming effects as its byproduct. In a basic spoofing attack type termed as “Meaconing”, the attacker simply captures the authentic GPS signals and re-transmit them towards the target. Also, an attacker could orchestrate a more advanced attack by constructing a fake GPS signal containing malicious information. Such attacks are termed as “Fabrication”.

Humphreys et al. (2008) groups the GPS spoofing attacks into three categories as (a) Simplistic, (b) Intermediate and (c) Sophisticated, based upon the complexity of the attack and the used hardware. *Simplistic* GPS spoofing is broadcasting arbitrary spoofed GPS signal without catering for the state of the targeted receiver. An *intermediate* GPS spoofing attack is centred on pre-surveyed information about the target such as publicly available parameters of authentic GPS signal being received by the victim receiver at the time of the attack. Lastly, a *sophisticated* attack uses multiple coordinated phase-locked intermediate spoofers to evade spoofing detection protocols of the target receiver.

The lack of any authentication mechanism makes the GPS receiver unable to distinguish between the authentic and malicious signal. Also, because of its open accessibility and publicly available technical parameters such as C/A code modulation, the Civil GPS can easily be mimicked using a signal simulator or low-cost open source equipment. On the contrary, replication of authentic P(Y) code used by the US Department of Defense (DoD) is technically infeasible due to its classified signal structure and limited information about the employed encryption technique.

A GPS spoofing attack can target to manipulate the PVT calculations at receiver end, either causing disturbances/deviations in time measurements or inducing errors in location measurements, as discussed in ensuing paragraphs.

#### Time spoofing

Spoofed GPS signal transmitted by an attacker can cause time-bias and abrupt changes in the victim’s receiver clock (Humphreys et al., 2008). In the case of a swarm of drones being controlled by a master drone, this type of attack will have catastrophic consequences as an alteration in the time of reference clock may induce errors in PVT calculations by the victim. Due to this clock offset, the master drone would be required to recalculate its position, which may lead it to a collision course with the slave drones within the swarm.

Apart from drones, time manipulation due to GPS spoofing also poses a serious threat to other time-dependent systems such as those used in the finance/banking sector, cellular communications, and energy distribution systems. The base stations (towers) of CDMA based communication systems also use GPS-reference time for the tower to tower communications. In a demonstration at the University of Texas, a 10*us* drift was introduced in CDMA-based cellular communications within 30 minutes of GPS spoofing attack, which resulted in disruption of CDMA communications (Humphreys, 2012). Similarly, Shepard et al. (2012) demonstrated the effectiveness of GPS based time spoofing attack against GPS time-reference receiver used by Power Measurement Units (PMU) in smart grid systems. The well-crafted spoofing attack induced a 400*us* of time drift that resulted in breaking the standard accuracy threshold of the measured phased angle recorded by the PMU. Furthermore, Wang, Chen & Pan (2015) demonstrated that a simplistic spoofing attack using an SDR can lead to time spoofing in a high-end smart-watch. Also, researchers in DEFCON 25 successfully demonstrated time manipulation by targeting Network Time Protocol (NTP) servers with a GPS spoofing attack (Karit, 2017).

To summarize, GPS spoofing based time manipulation attacks can affect the perceived time of GPS devices resulting in erroneous path planning and collision of aerial platforms, impairing cellular communications and having the potential to cause blackouts due to failure of the power distribution systems because of their dependency over GPS-time.

#### Location spoofing

**Figure 4:**
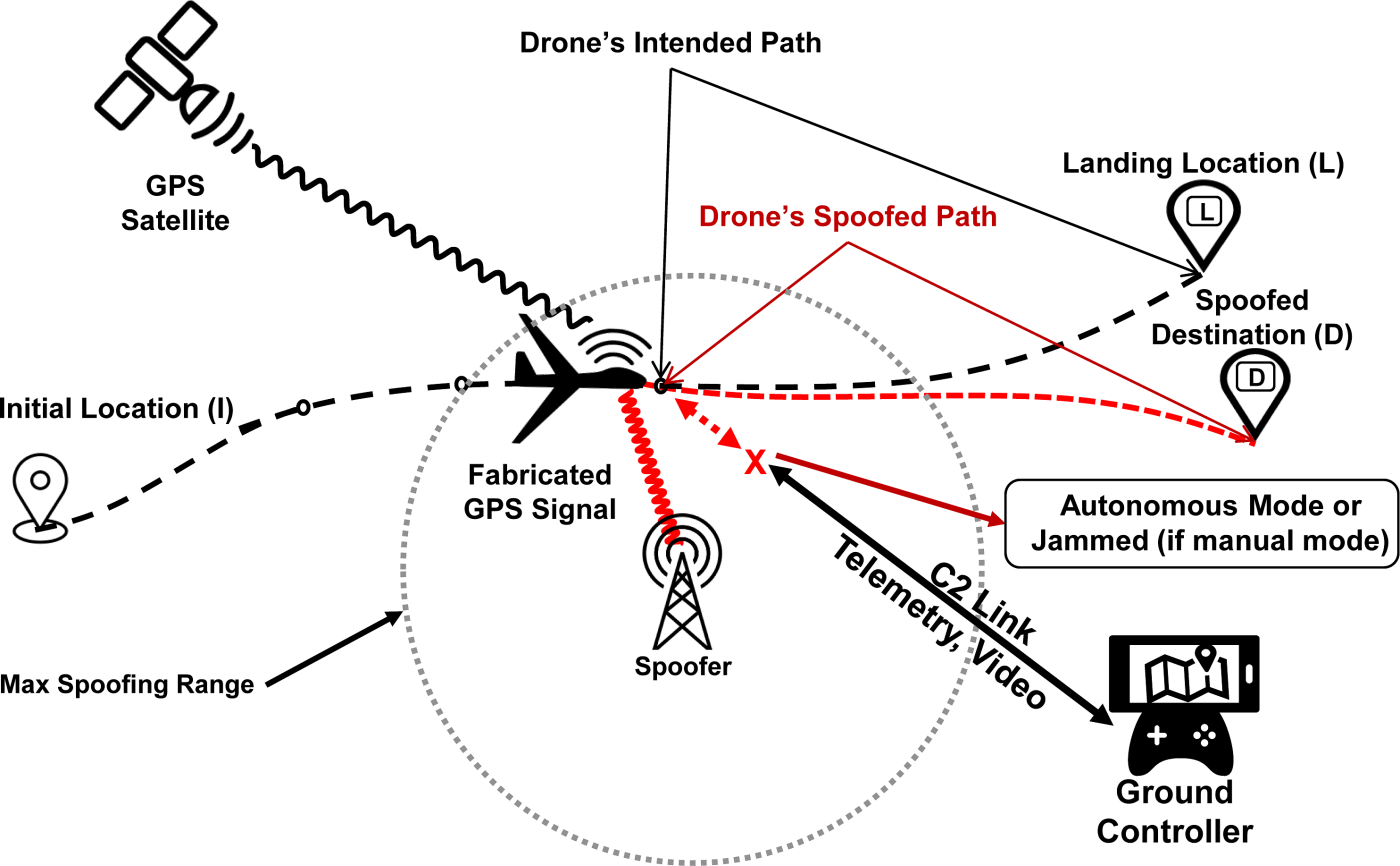
GPS spoofing of UAV: an illustration.

Fundamentally, a GPS spoofing attack results in manipulating the target’s GPS based location calculations by inducing an inaccurate position fix, as depicted pictorially in Fig. 4. As covered in section ‘Drones GNC Dependency and Allied Threats, drones rely on GPS for navigation and positioning in different modes. This dependency makes them vulnerable to location spoofing attacks. With the ever-increasing acceptability and adoption of autonomous GPS-driven traffic management systems, such as NEXT GENeration air-traffic system (NEXTGEN) by the USA, GPS location spoofing threats are becoming even more realistic, having a detrimental impact on the safety of such systems (Schmidt et al., 2016). In the recent past, several research efforts have successfully demonstrated the applicability of GPS location spoofing against commercially available drones. In an indoor environment Arteaga et al. (2019), successfully spoofed the location of 3DR Solo drone using an SDR device running an open source script. Similarly, Dey et al. (2018) and Horton & Ranganathan (2018) demonstrated that even the sophisticated consumer drones from DJI are susceptible to simplistic spoofing attacks and can be easily tricked by fabricated GPS signal in absence of the authentic GPS signal. Also, spoofing the location of a drone can result in diversion from the actual course, crashing, hijacking, or even gaining full control of the target, forcing it to land at a place of attacker’s choice. Ma et al. (2020) demonstrated that drone in *autonomous mode* can be lured to a pre-defined destination by executing a GPS course deviation attack. Using GPS spoofing as a defensive tool against intruding GPS dependent drones, He & Liu et al. (2019); He & Qiao et al. (2019) demonstrated path deviation attacks by inducing velocity drifts in *loiter mode* of the drone.

Apart from aerial platforms, GPS is also used extensively in other areas for tracking and location information e.g., shipping, trains, cargo trucks, and taxis. Commercial truck drivers can use GPS based location spoofing to follow unauthorized routes or assist in fraudulent theft of truck consignments or the truck itself (Schmidt et al., 2016). A similar off-board attack setting was demonstrated by Warner & Johnston (2002), simulating falsified location information reported by cargo trucks. Zeng et al. (2017) demonstrated a location spoofing attack in a road navigation scenario using an SDR based spoofing device. Zeng et al. (2018) generated hundreds of ghost road routes in Boston and Manhattan, USA, that could be used to divert, endanger, or even hijack the victim vehicle.

#### Generation of spoofing parameters using SDRs

Beside specialized hardware available for simulation and testing of GPS signal such as Labsat GNSS Simulator (https://www.labsat.co.uk/), SDRs are now being extensively used for the generation of spoofed GPS signals due to their easy operation, low-cost and open source availability of supporting codes. Many of the GPS spoofing projects available in literature leverage an open source GitHub project by Ebinuma et al. (https://github.com/osqzss/gps-sdr-sim) for launching simplistic GPS spoofing attacks (Hermans & Gommans, 2018). Gaspar et al. (2020) utilised BladeRF, a low-cost SDR device, to generate spoofed GPS parameters for deviating the path of a targeted drone.

The source code in many of the successful projects benefits from the openly accessible ephemeris files by NASA’s data archive. These published ephemeris files by Crustal Dynamics Data Information System (CDDIS) (https://cddis.nasa.gov/archive/gnss/data/daily/) contains anticipated or extrapolated future information of GPS satellites’ orbits that are only valid for a few hours. A fresh set of ephemeris data can also be obtained by decoding navigational message received directly from satellite (Huang & Yang, 2015). Using the information obtained through these ephemeris files, a malicious locus GPS signal is crafted for spoofing of the target to the desired point. Based on the given conditions, spoofed GPS signals are generated as a solution for the trilateration equation listed in ‘Background’. Subsequently, In-phase/Quadrature (I/Q) data is generated for the modulated pseudo GPS signal and transmitted at given L1 (C/A) frequency of GPS with a sample rate of 2.6 MHz (Hermans & Gommans, 2018).

## Taxonomy of GPS spoofing attacks

**Figure 5:**
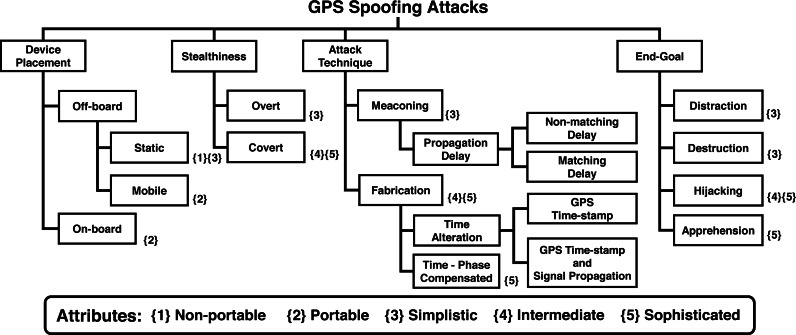
Taxonomy of GPS spoofing attacks.

We present a novel taxonomy of GPS spoofing attacks after categorizing them based on different parameters, including location of the spoofing hardware, attack stealthiness, attack methodology/technique, and the end-goal of the spoofer. The overall taxonomy is depicted in Fig. 5, while the details of its design parameters are discussed in subsequent paragraphs.

### Device placement

The spoofing hardware, coupled with the algorithm used for GPS spoofing, jointly determine the attack’s effectiveness. Researchers have demonstrated spoofing using a diverse range of devices, ranging from Commercial Of The Shelf (COTS) based low-cost equipment to custom-built sophisticated spoofing systems. The selection of an appropriate spoofing hardware depends on a variety of factors, including the relative location and velocity of victim receiver, affordable cost, link-budget analysis (requirement of dedicated signal amplifiers), terrain (requirement of a clear line of sight), attack directivity (to minimize collateral damage), and embedded anti-spoofing capabilities of the target. Based upon these key parameters, spoofer’s placement can be classified into Off-board and On-board spoofers, as discussed below.

#### Off-board

Spoofing devices that are kept at a distance from the target. *Off-board spoofers* can be both “Static” or “Mobile”.

##### Static spoofer.

Static spoofer refers to the spoofing equipment that is non-portable and usually ground fixed. Generally, static GPS spoofers include GPS transmitters that are bulky, immobile, non-tunable and hardware-based. However, because of their static nature, these spoofers can afford high power amplifiers, which can help them generate strong spoofing signals to compromise distant receivers as well. A diverse range of static GPS emulators/spoofers are available in the market which can be graded based on their capabilities, such as the number of satellite signals which can be generated simultaneously, level of programming and control offered over signal generation, and multipath modeling/compensation features. As an example, the static spoofing hardware (WelNavigate GS72) used by Warner & Johnston (2002) was able to simulate only 10 satellites signals at a time as compared to the currently available high-end simulators that can simulate up to 64 simultaneous signals and multiple GNSS systems e.g., the Orolia GSG-5/6 series GPS simulators (https://www.orolia.com/products/gnss-simulation/gpsgnss-simulators).

##### Mobile spoofer.

Technological advancements in recent years have facilitated the hardware-specific equipment capabilities to be achieved by flexible and user-friendly software modules, thus significantly reducing the SWaP-C parameters, resulting in low cost and user-friendliness. The SDRs, such as HackRF One, BladeRF and USRP mini are portable palm-size frequency tunable devices that are capable of generating GPS spoofing signals. Due to their mobility and ease of use, SDRs are now widely being deployed for GPS emulations and offensive transmissions. A number of recent research efforts (Shepard et al. (2012); Dey et al. (2018); Horton & Ranganathan (2018); Arteaga et al. (2019); He & Qiao et al. (2019); He & Liu et al. (2019); Ma et al. (2020)) have demonstrated GPS spoofing attacks against several drones using low-cost SDR equipment and open source scripts. Moreover, practical demonstrations of GPS location spoofing attacks against non-aerial platforms using commonly available SDR devices have been put forward (Huang & Yang (2015), Wang, Chen & Pan (2015); Silva (2017), Zeng et al. (2017), Horton & Ranganathan (2018), Zeng et al. (2018), Goavec-Merou, Friedt & Meyer (2019) and Gaspar et al. (2020); Rustamov et al. (2020)). Similarly, GPS based time spoofing attacks using SDRs have been demonstrated by Shepard et al. (2012); Wang, Chen & Pan (2015); Karit (2017).

**Figure 6:**
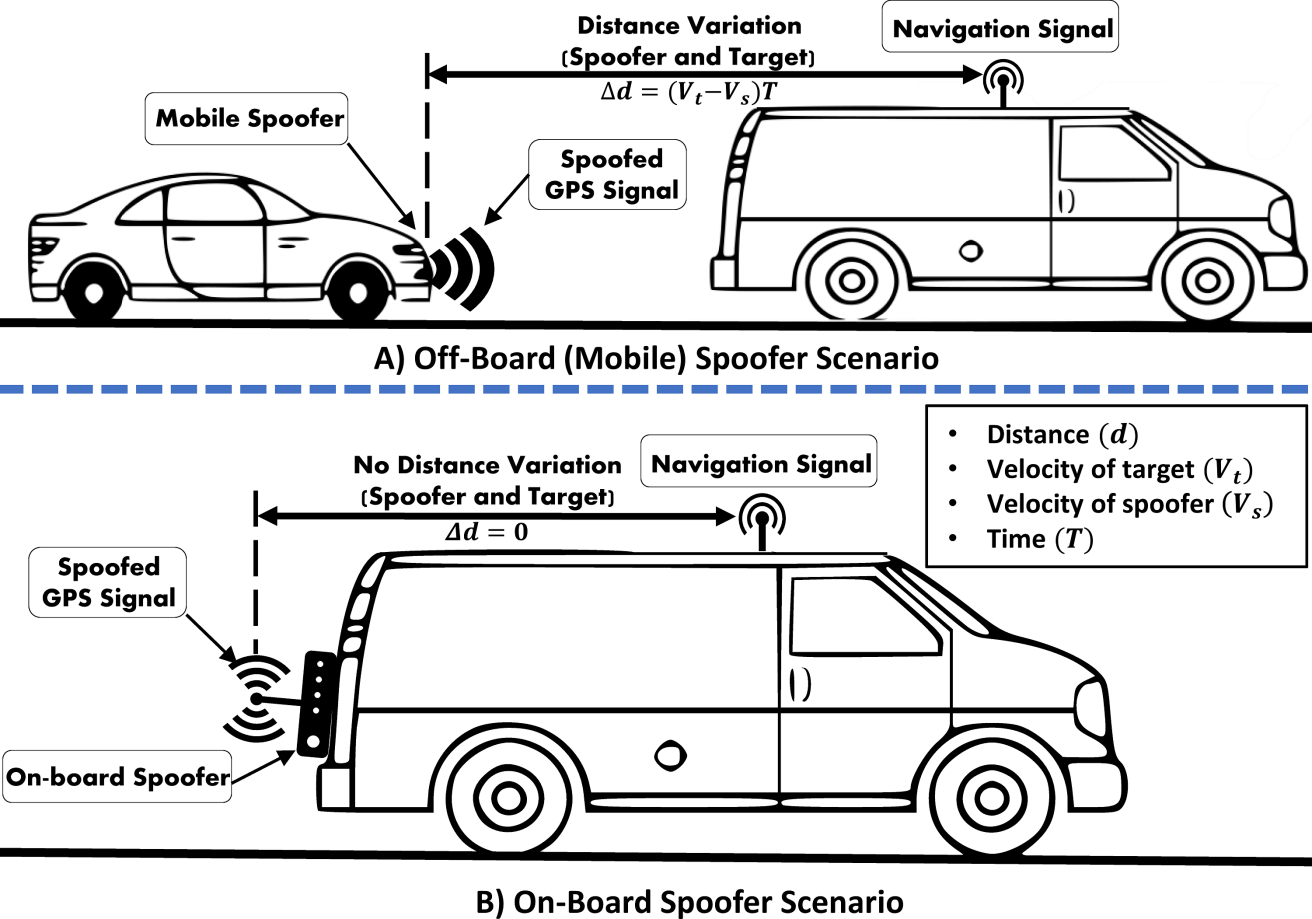
Off-board/On-board GPS-location dependent vehicle spoofing scenarios.

Using an off-board spoofer against a moving target have its own challenges. The key challenge is to consistently and accurately maintain the desired signal strengths and phase angle required to spoof a fast moving/flying receiver. As depicted in Fig. 6A, the variation in distance (Δ*d*) between the target receiver and the off-board spoofer is a function of difference in their individual velocities and time. These distance variations induce abrupt fluctuations in RSS at the victim GPS receiver, which can be used to filter out the spoofed GPS signals.

#### On-board

GPS spoofing systems that are covertly attached to the target are known as *on-board spoofers*. A miniature size one-board spoofer, also known as a Limpet spoofer, is a portable device with an independent power source and is normally concealed at a suitable location over the target platform to safeguard its operations (Lo & Enge, 2010). *On-board* or *limpet spoofer* normally requires a wireless communication link with its base controlling station to receive spoofing commands and send feedback. Additionally, it can also be pre-programmed and configured for autonomous operations. However, autonomous spoofers lack the desired flexibility and are, therefore, deemed unsuitable for spoofing mobile platforms, especially when prior knowledge of mission trajectory/path is not available.

Not much work can be found on the use of limpet spoofer against aerial platforms. Zeng et al. (2018), used a HackRF One based limpet spoofer, which was installed over the victim’s vehicle as depicted in Fig. 6B.

On-board location of the spoofing equipment can simplify many challenges in spoofing attacks such as inducing the required delay and phase angle for a moving target. For an off-board apparatus, the attack’s complexity increases manifold, as such a spoofer is required to cater for the variations in the distance and angle from the mobile target. As an example, Shepard et al. (2012) used an off-board spoofer to spoof a Hornet mini drone and compensated for the signal variations by relying on the perceived position of the target. However, Zeng et al. (2017) successfully spoofed the actual position of GPS-enabled vehicles using an on-board spoofing device, as shown in Fig. 6B. Likewise, in Bhatti & Humphreys (2017), a 65 m yacht was set 3-Degrees off-course using an on-board custom-made GPS spoofing set-up.

On the other hand, mounting of spoofing device on the victim’s GPS receiver requires physical access to the victim’s platform, which might not be possible in most of the hostile situations. Moreover, since the access and movement of the victim’s platform are not under spoofer’s control, physical security, remote programmability, and reliable connectivity to the spoofer are other key challenges, hindering the safe and effective operations of an on-board spoofer.

### Stealthiness

Based on the stealthiness and strategy of the attack, GPS spoofing attacks can be divided into two broad classes:-

• **Overt:** The spoofer does not attempt to obscure the attack.
• **Covert:** The spoofer seeks to evade detection by transmitting smartly-crafted spoofing signals, which closely match the actual satellite signals in terms of output power and other parameters. By doing so, the spoofer prevents triggering of spoofing detection alarm by the victim.

In an overt spoofing attack, the victim GPS receiver loses lock on the authentic GPS signal before switching to the over-powered spoof signal. The strategy of jam-then-spoof is adopted in such overt scenarios, which leads into abnormally high SNR followed by an abrupt jump in PVT solution calculated by the victim (Chapman, 2017). The overt spoofing also termed as Hard spoofing (Noh et al., 2019) can be easily detected due to signal interruptions in the initial phase and abnormally high SNR (Gao et al., 2013). Some rudimentary anti-spoofing checks can be implemented to detect such types of GPS spoofing attacks by analyzing the position solution and GPS signal’s strength; however, most civil drones still lack these basic defenses due to simplistic and security unaware designs (Humphreys et al., 2008). Compared to the overt attack, a covert spoofing attack is an advanced level and sophisticated operation. In a covert attack, drifts in velocity and positioning values are induced in a concealed manner to enforce the target to follow a spoofed path that may result in capturing the target. Su et al. (2016) proposed a greedy strategy of covertly spoofing UAVs, causing them to deviate from the intended flight paths. The proposed approach assumed a capability to generate code and phase-aligned spoofed signal with reference to the authentic GPS signal. It subsequently applied the minimum malicious deviation of the target as an expanding location range circle over time without triggering the detection alarm. The experimental results by Tippenhauer et al. (2011) presented a set of threshold parameters to successfully execute covert spoofing against advanced GPS receivers. The authors concluded that for seamless lock take over by a spoofer, a minimum of 2 dB power advantage is desired, while a maximum of 80 nsec of time offset relative to the authentic signal and 500 m location offset is to be ensured to remain undetected. Peng et al. (2019) concluded that for a covert attack, the spoofing signal must have a maximum power advantage of 3 dB, while a maximum carrier frequency offset of 50 Hz can be afforded. Another approach towards stealthy spoofing was proposed by Gao et al. (2013), which presented two different two-step trajectory spoofing strategies. In the first step, the attacker maintained very low power for the spoofing signal and carefully aligned the code phase of the transmitted signal similar to the authentic signal. In the second step, the spoofer attempted to isolate the tracking point induced by the spoofing signal from that of the genuine signal. Psiaki & Humphreys (2016) argued that attacker may adopt advanced spoofing forms such as nulling and multi-antenna spoofers, for covert spoofing and defeating various defences deployed by the victim GPS receiver. In nulling, two simultaneous signals including a true spoofed signal and a negative authentic signal are transmitted so that the negative signal cancels the authentic signal due to carrier phase shift. Similarly, a multi-antenna spoofer, with single or multiple transmitters, can deceive some advanced anti-spoofing countermeasures by implementing independent delay variations, multiple steerable gains, and scattered/controlled direction of arrival of spoofed signal.

### Attack Technique

The technique deployed for GPS spoofing depends upon several factors such as spoofer’s hardware capabilities, algorithm’s sophistication, and the information available with the spoofer about victim’s parameters, such as its real-time location, velocity, antenna placement, and anti-spoofing features. Based on the attack technique used, GPS spoofing attacks can be classified as:-

#### Meaconing

Meaconing is defined as the re-radiation or replaying of the original GPS signal by intercepting and then rebroadcasting it for the malicious purpose of confusing the GPS receiver by causing time-drift (Panice et al., 2017). Meaconing, also termed as “replay attack” is a fundamental type of spoofing. For an attacker, meaconing attack is easy and equally applicable to the civil and military GPS signals since the attacker is not required to decrypt the encrypted *P*(*Y*) code. However, the meaconing spoofer is limited to controlling the signal’s delay only and cannot apply modifications to the signal’s parameters (Günther, 2014).

##### Propagation Delay.

In this attack type, the attacker generates the spoofed GPS signal with customized signal propagation delay by transmitting earlier or after the original GPS signal, while keeping the authentic GPS time-stamp unchanged. The spoofer is capable of adding fixed or varying signal propagation delays for an individual satellite in the spoofed signal.

• **Matching Delay:** The attacker fixes a constant delay value for all satellites that constitute the spoofed signal.
• **Non-matching Delay:** The signal propagation time of each satellite signal is manipulated independently by introducing non-matching/unequal delays in the spoofed signal.

#### Fabrication

A more advanced type of GPS spoofing is the generation and transmission of fabricated GPS signals to deceive a GPS receiver, forcing it to execute desired malicious commands that may result in gaining complete control of the system. As compared to GPS jamming and meaconing, full reconstruction of the GPS signal is an advanced level attack. For such an attack, a false GPS signal having spoofed information of the almanac and ephemeris parameters is transmitted in the direction of the GPS receiver with a power advantage, forcing it to synchronize with the spoofed signal. If the targeted UAV’s GPS receiver switches form the original GPS signal to the fabricated GPS signal, then the spoofer can potentially deceive the victim. For an enhanced GPS spoofing attack, various other requirements such as calculation of the spoofed location by the attacker and directivity of the spoofing signal for a targeted attack, are explained in Tippenhauer et al. (2011) and Hermans & Gommans (2018), respectively. Similarly, Renyu et al. (2018) presented provisions of spoofing attacks using an array of GPS-guided drones.

#### Time alteration

An attacker can manipulate the time of the spoof GPS signal by changing the GPS time-stamp or varying the propagation time of the signal and the GPS time-stamp simultaneously (Wei & Sikdar, 2019).

##### GPS Time-stamp.

The attacker generates the spoof GPS signal with GPS time-stamp that is different from the authentic GPS signal while keeping the signal propagation time unchanged as of the authentic signal. Altering the GPS time-stamp results in affecting time and location perceived by the target receiver.

##### GPS time-stamp and signal propagation.

In this type of attack, the spoofer generates the spoofed GPS signal by manipulating both the GPS time-stamp and signal propagation time simultaneously.

#### Time and phase compensated attack

This is a sophisticated attack category in which the attacker has complete knowledge of the target’s location and its antenna placement. Considering the position and location of the target’s antenna, the spoofer generates a spoofing signal with a systematic delay and phase angle.

#### Analysis of attack techniques

Regarding the attack technique used by the spoofer, compared to fabrication the meaconing or replay attacks are simplistic in nature and often work well in scenarios where the target is stationary or slow moving, e.g., cell phone, as demonstrated by different research efforts (Warner & Johnston, 2002; Huang & Yang, 2015; Wang, Chen & Pan, 2015; Silva, 2017; Horton & Ranganathan, 2018; Goavec-Merou, Friedt & Meyer, 2019; Gaspar et al., 2020; Rustamov et al., 2020). For a covert attack against a moving target, either the spoofing equipment is to be on-board i.e., attached to the target or real-time location of the target should be known to the spoofer so that an appropriate phase angle and time delay can be induced in the spoofed signal (Humphreys, 2012). The spoofing techniques presented by Zeng et al. (2018) and Horton & Ranganathan (2018) required prior knowledge about the intended route of travel and home location of victim’s receiver, respectively, for the successful execution of their spoofing algorithms. In case of UAVs, the ADS-B broadcast of the aircraft can also be tapped to determine its location (Kerns et al., 2014; Naeem et al., 2021). Against other moving platforms such as GPS guided vehicles, different novel approaches have been introduced (Warner & Johnston, 2002; Tippenhauer et al., 2011; Humphreys, 2012, Wesson & Humphreys, 2013) for mitigating such spoofing challenges.

As for the effectiveness of attack techniques, Wei & Sikdar (2019) demonstrated that if only the pseudo-range error is considered by the victim, the *matching delay* attack is difficult to perceive due to its negligible error. On the contrary, *non-matching delay* attack due to its large pseudo-range errors can be easily detected by the victim. Similarly, *GPS time-stamp* and *signal propagation* attack can also be easily detected as it results in large pseudo-range errors calculated by the victim. However, attacks by altering the GPS-Time stamp only are difficult to be detected and distinguished from normal interference due to negligible deviations in time as recorded by the victim.

### End-goal

The GPS based spoofing attacks against moving targets, specifically UAVs, can also be categorized based on the attacker’s objective. An attacker may seek diverse end-goals from spoofing attempts, such as distraction, destruction, endangering, and apprehension of the victim’s platform (Giray, 2013; Zeng et al., 2018). Achievement of the attacker’s end-goal largely depends upon the spoofer’s capabilities vis-a-vis victim’s anti-spoofing features. A spoofer can attack a GPS-guided aerial platform to achieve the following end-goals:-

• **Distraction:** Randomly *location spoof* the target with an aim to prevent or delay it from reaching its destination.
• **Destruction:** Endangering the target by setting it on a collision course, either towards an aerial obstacle or hitting the ground through manipulation of its height parameters.
• **Hijacking:** Gaining a temporary control of its target to usurp the victim.
• **Apprehension:** Directing the victim to a pre-defined destination and then forcing it to safely land inside a friendly zone for capturing the drone or its payload.

Various types of similar spoofing attacks against drones can be found in He et al. (2019); Shepard et al. (2012); Ma et al. (2020); Horton & Ranganathan (2018). In He et al. (2019) a low-cost capturing attack technique against a drone in RTL mode was presented. A simplistic location spoofing attack can be deployed in RTL mode, causing the victim to retrieve, distract, or even suffer damage/destruction. Also, a GPS-time spoofing attack may also lead to distraction and destruction in case of UAVs and smart-grid systems. A *hijacking* attack against a GPS dependent mini-drone was demonstrated in Shepard et al. (2012) by gaining an interim control over the victim. Ma et al. (2020), managed to lure a DJI Phantom 3 SE drone to a pre-designated spoofed location at a distance of about 50 *m* from its actual destination. Similarly, Noh et al. (2019) classified various consumer drones based on their GPS fail-safe mechanisms and presented three different GPS spoofing-based hijacking strategies: (a) inducing drift in a specific direction, (b) manipulation of the trajectory using a path-following algorithm, and (c) combination of (a) and (b) through covert spoofing. Furthermore, all the above-stated attack objectives can also be achieved against ground-based targets such as GPS-driven autonomous vehicles. As an example, Zeng et al. (2018) demonstrated spoofing attacks against a road navigation system to achieve different objectives such as *Diversion*, *Distraction*, and *Apprehension*.

## Spoofing challenges

### Relative position of the spoofer

The effectiveness of a GPS spoofing attack, besides other factors, relies heavily on the relative position of the spoofer with reference to the victim receiver. This factor alone induces the peculiar challenges of spoofing a ground-based target in comparison to an airborne platform. Spoofing of a ground-based receiver requires establishing a consistent and clear line of sight with the victim, which becomes difficult to manage, particularly against a moving vehicle. Ground spoofing also requires sophisticated algorithms to compute alternate routes (in case of complex and congested road networks) to effectively achieve the *Diversion* or *Distraction* goals, without getting detected. As an example, Zeng et al. (2018) proposed novel diversion algorithms for ground-based spoofing scenarios, which also catered for practical road turnings/branches at the city level to make them effective/covert. This challenge is not particularly relevant for spoofing of aerial platforms as they can be flexibly *Diverted*/*Distracted* in the open 3D flying environment. Moreover, the desired line of sight required for the spoofing attack is also easier to establish for aerial platforms due to lesser obstructions in free space. Another worth-mentioning challenge for a ground spoofing scenario under a dense user environment is maintaining the desired directivity towards the intended receiver(s) to avoid collateral damage to other receivers operating nearby. This limitation may also be relevant for airborne scenarios when only a given drone is to be spoofed within a swarm or when friendly airborne assets are operating in close vicinity to the targeted hostile drone.

### Spoofer’s distance variations

UAVs typically fly at considerably high altitudes and operate over a wide range of speeds, reaching as high as the speed of sound. These flight profiles significantly add to the complexity of ground-based spoofing attempts. The rapidly changing distances between the airborne target and ground-based spoofer can cause an abrupt fluctuation in the strength of the spoofer’s signal as received by the victim, as per the free space square law:

\begin{eqnarray*}Pr=Pt/4\pi {d}^{2} \end{eqnarray*}

Where *Pr* is the received power, *Pt* is the power transmitted by the system and *d* is the distance between the two antennae. As the above formula suggests, the received power varies inversely with the square of the distance between the two transceivers. For the spoofing attempt to go undetected against modern-day sophisticated receivers, the transmitter and receiver must maintain a fix distance or power ratio. For a drone, the distance between the on-board GPS receiver and GPS satellites is insignificantly varying as the satellites are hundreds of kilometers above, so the power remains relatively constant. Therefore, for a successful spoofing attack against an airborne platform, particularly if operating beyond a controlled territory, maintaining a constant signal strength is one of the core challenges. Many research efforts (Jafarnia-Jahromi et al., 2012; Schmidt et al., 2016; Mathew, 2019) endorse that spoofing a mobile GPS receiver with sustained/constant power suffers this practical limitation due to abrupt distance variations between the spoofer and victim GPS receiver.

### Spoof resistant receivers

Besides the relative position of spoofer, the anti-spoofing capabilities of the victim receiver also impact the effectiveness of the attack. These spoofing countermeasures have been extensively discussed in literature (Key, 1995; Humphreys et al., 2008; Wesson, Shepard & Humphreys, 2012; Habib, Maqbool & Mohsin, 2019) and warrants careful analysis of the targeted receiver to customize the corresponding spoofing parameters. Ranganathan, Ólafsdóttir & Capkun (2016) presented a spoof resistant GPS Receiver capable of receiving both strong and weak GPS signals and tracking any auxiliary peaks to detect sophisticated spoofing attempts capable of seamless lock takeover of GPS receiver. Arthur (2019) proposed an Artificial Intelligence (AI) based Intrusion Detection System (IDS) against GPS jamming and spoofing attacks on UAVs. Similarly, another AI-based supervised machine learning method was proposed by Manesh et al. (2019) for the detection of counterfeited GPS signals. This approach leveraged different GPS signal parameters as input features for the neural network, including satellite number, SNR, pseudo-range, doppler shift, and carrier phase shift and tried different combinations of these features to analyze the accuracy and false alarm rate of achieved results. Furthermore, Eldosouky, Ferdowsi & Saad (2019) proposed a framework for modeling the optimal flight route of a UAV as a defence mechanism to mitigate the effects of a GPS spoofing attack on UAVs in *autonomous mode*. Naeem et al. (2021) presented a novel obfuscation-based approach to safeguard location parameters against GNSS spoofing by concealing actual location coordinates or intentionally deceiving a known adversary by sharing wrong coordinates, thus averting eavesdropping of UAV’s actual trajectory which is an important information needed for covert spoofing attacks. Furthermore, Oligeri et al. (2019) presented a GPS spoofing detection and mitigation mechanism by leveraging broadcast signals of reference cellular network for validation of location measured through GPS infrastructure. The proposed solution is feasible for GPS devices with cellular connectivity such as smartphones and road navigation systems and is not applicable over UAVs.

### Multi-GNSS receivers

Multi-GNSS receivers are capable of providing accurate positioning and navigation solution by simultaneously utilizing two or more GNSS systems i.e., GPS, GLONASS, and BDS etc. For example, GN-87 is a Multi-GNSS receiver, capable of concurrently receiving signals from GPS, Galileo, and GLONASS (FURUNO Electric CO., 2019). It can switch its operations to another GNSS system in case of attempted jamming and spoofing attack or unavailability of the GPS signal, making it challenging for the spoofer to deprive or manipulate the UAV’s GNSS-dependent services. Currently, a number of high-end UAVs in the market are equipped with multi-GNSS receivers (DJI, 2020).

### Angle of Arrival (AoA) of signal

GPS antenna is typically mounted on top of a UAV to have a clear line of sight with GPS satellites. A ground-based spoofer will find it hard to direct its transmissions towards the victim’s antenna. Another potent anti-spoofing measure for UAVs is to filter out fake GPS signals or ground reflections by equipping the on-board GPS receiver with Direction Finding (DF) capabilities. Since many spoofers generate multi-satellite signals from a same source, such signals received from a single location can be filtered out or rejected by the direction finding system. A successful spoofing attack under such a scenario would require an exact location of on-board GPS antennae and corresponding direction finding algorithm used by the victim to generate precise delays and phase shifts.

Montgomery (2011) demonstrated the effectiveness of AoA technique as a GPS spoofing countermeasure and claimed its effectiveness against sophisticated spoofing attacks. In the case of a group of drones, considering the required phase angle for multiple victims, the spoofer is restricted to fewer spoof location choices with the increase in the number of subjects (Tippenhauer et al., 2011).

## Open Problems and Future Research Directions

GPS has been a tempting target for security researchers due to its widespread applications and inherent vulnerabilities. This section highlights some of the open problems within the domain of GPS spoofing and points to future research directions to motivate further contributions.

**UAV Spoofing Using Follower Spoofers:** Study of spoofing constraints using airborne follower/limpet spoofers is an interesting research direction as not much work could be found in this domain. Limpet spoofers have tight SWaP-C constraints and if realized, they can be deployed as hostile follower drones or “unfaithful wingmen” to maintain a constant distance and angle from the victim drone. While this approach can simplify spoofing algorithms by eliminating range and angle variation parameters, it induces new research challenges, such as remote control of follower UAV spoofer and reliably achieving the follower trajectory without any sensory/trajectory assistance from the victim UAV.

**Spoofing Multi-GNSS Receivers:** Another open research problem is to explore the possibility of spoofing those UAVs which are equipped with multi-GNSS receivers. This can be achieved by the simultaneous use of multiple synchronized spoofers, each targeting a specific GNSS receiver, either through predefined (fixed) or adaptive (dynamic) spoofing parameters, tailor-made for that receiver. Some of the worth-mentioning challenges for such an arrangement include inter-spoofer synchronization, interference, power, and directivity management.

**SWaP-efficient DF for UAV Deployment:** As already discussed in ‘Spoofing Challenges’, equipping UAV’s GPS navigation system with DF capability can help in detecting and rejecting spoofing attacks. Development and testing of SWaP-efficient DF systems, which could be integrated with GPS systems on-board lightweight aerial platforms is another open research problem.

**Obfuscation-resilient spoofing algorithms:** Location obfuscation techniques, such as proposed by Naeem et al. (2021), can be deployed to defeat spoofing algorithms. Another interesting research direction is to develop obfuscation-resilient spoofing algorithms, which are capable of decoding obfuscation parameters and counter them through appropriate spoofing techniques. This domain requires investigating into spoofing strategies which can effectively spoof UAVs with inaccurate/incorrect location information.

## Conclusion

With the dawn of smart robotics, intelligent sensor fusion, and the IoT, the modern world is witnessing exponential growth and wide acceptability towards autonomous systems. GPS-driven GNC applications serve as a valuable enabler to realize such systems. However, the inherent vulnerabilities of GPS-based services pose serious security threats, including location and time spoofing of safety-critical dependent applications.

In this paper, we presented a comprehensive review and critical analysis of existing efforts towards GPS spoofing. In particular, location spoofing of UAVs was covered in detail by correlating GPS dependency with UAVs’ operational modes and analyzing attack variations for static, limpet, and mobile (follower) spoofers. An attacker can deviate, jeopardize, destroy, or even hijack a spoofed UAV with the help of well-crafted fabricated GPS signals. We also presented a novel taxonomy to classify attack capabilities, location, stealthiness, and objectives of multifaceted spoofing techniques, while grouping and discussing the available literature as per the definitions of our taxonomy. The paper also covered some of the open problems to motivate further research in focused directions. A review of existing literature reveals diverse GPS spoofing attacks against aerial-platforms, surface vehicles, and other statics services, calling for design of security-aware and spoof-resilient GPS services. On the other hand, GPS spoofing has also shown promising potential for parametric defence to neutralize hostile drones.

## Additional Information and Declarations

### Data Availability

The following information was supplied regarding data availability: The research is a survey with no raw data or code.
